# Local and global response data from post-fire earthquake simulations of RC structural walls

**DOI:** 10.1016/j.dib.2018.06.011

**Published:** 2018-06-13

**Authors:** Shuna Ni, Anna C. Birely

**Affiliations:** Zachry Department of Civil Engineering, Texas A&M University, United States

## Abstract

Data are provided from simulation studies of post-fire earthquake (PFE) of reinforced concrete (RC) structural walls. Local response data describe material peak temperatures and residual stiffness and strength, as well as quantification of the extent of seismic damage. Global response data provide load-deformation envelopes, as well as stiffness, strength, and deformation capacity. The data can support development of simplified modeling tools for PFE analysis of buildings, as well as to support development of and interpretation of future experimental tests. For further theory behind the modeling approach and full interpretation of the data, the reader is referred to the article entitled “Post-fire seismic behavior of reinforced concrete structural walls” (Ni and Birely, 2018) [1].

## Specifications Table

**Table:** 

Subject area	*Engineering*
More specific subject area	*Structural Engineering*
Type of data	*Tables, Figures, Text, Graph*
How data was acquired	*Simulation models*
Data format	*Filtered and analyzed*
Experimental factors	*Simulations of planar reinforced concrete walls subjected to post-fire earthquake (PFE) loads. Thermal analysis was conducted using SAFIR. Seismic analysis was conducted using OpenSees.*
Experimental features	*Walls are rectangular walls with flexure-controlled response. Reference wall is representative of typical characteristics of buildings on West Coast of the United States. Additional walls vary characteristics typically considered to impact fire and/or seismic resistance.*
Data source location	*Simulations were run on supercomputer resources at Texas A&M University, College Station, TX.*
Data accessibility	*Data provided in this article is accessible to the public. Input and output files for simulations are available at the DesignSafe-CI* (www.designsafe-ci.org) *data repository:*10.17603/DS22T12

## Value of the data

• Data can be used to develop methodology for simplified analysis of post-fire earthquake (PFE) of reinforced concrete (RC) walls.
• Data can be used to guide design of experimental test programs for PFE behavior of RC walls.
• Data can be compared with future experimental tests and/or more detailed simulation models to advance state-of-knowledge of the response of RC walls to PFE, particularly stiffness, strength, drift capacity, and failure mechanisms.

## 1 Data

This paper provides local and global response data for a parameter study on the post-fire earthquake response of flexure-controlled planar reinforced concrete structural walls. Local data include temperature, residual material properties, and an indicator of seismic damage. Global data include load-deformation envelopes and response quantities describing the behavior.

Background, results, and interpretation of the parameter study are described in [1]. A full description and validation of the simulation approach is described in [2]. Section 2 provides an overview of wall characteristics and applied loads. Section 3 describes local response data. Section 4 describes global response data.

## 2 Wall characteristics and loading

**Fig. 1:**
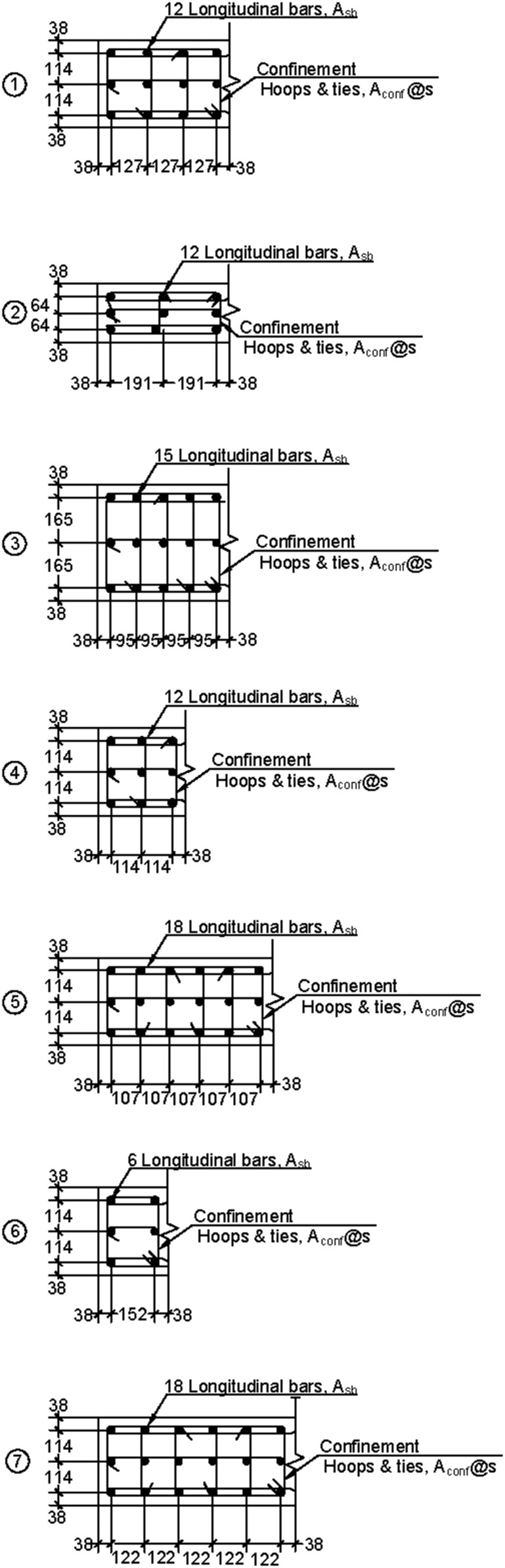
Boundary element reinforcement configurations.

**Fig. 2:**
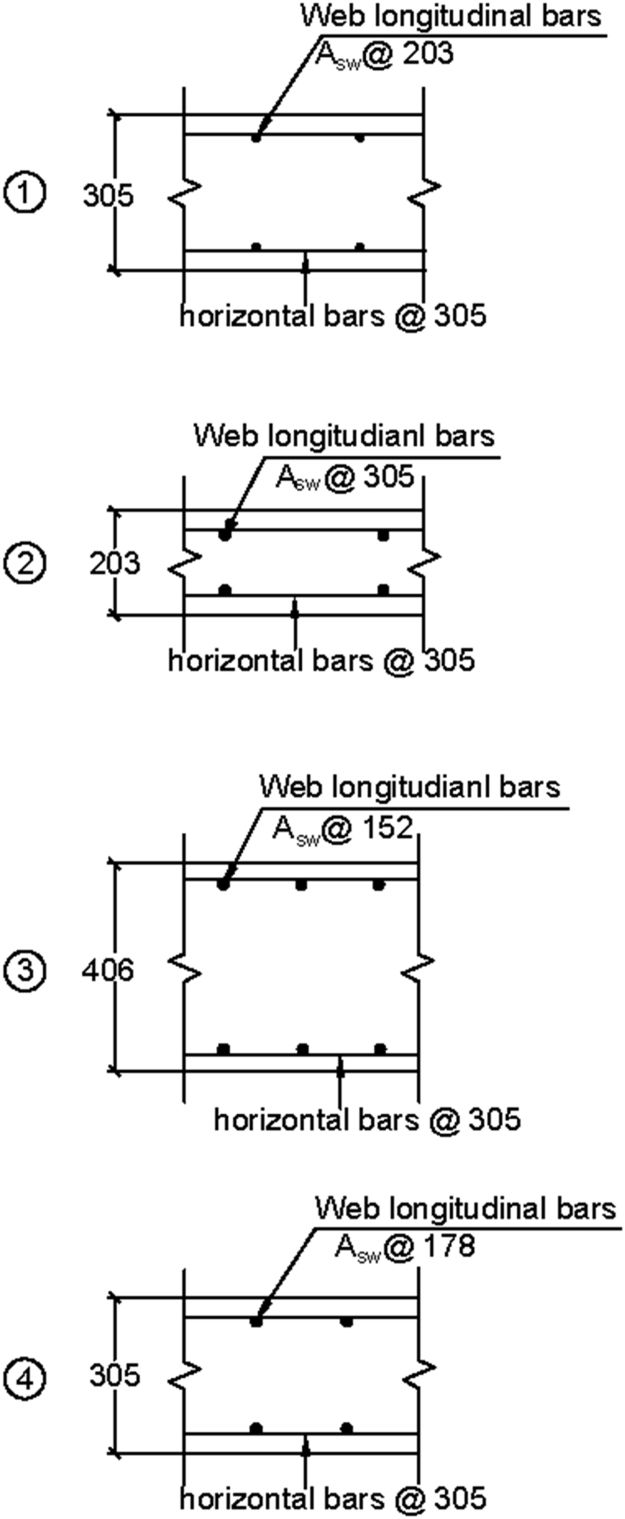
Web reinforcement configurations.

Twenty-one unique reinforced concrete planar walls were modeled. Wall 1 is the reference wall, with all other walls varying a single characteristic from the reference wall (geometry, reinforcement, or axial load). Table 1 provides wall geometry and axial load. Table 2 provides wall reinforcement with boundary and web reinforcement information. Boundary and web reinforcement detailing are shown in Figs. 1 and 2, respectively.

**Table 1:** Wall geometry and axial load.

	**Wall ID**	**h**_**w**_	**l**_**w**_	**t**_**w**_	$l_{\mathrm{be}}/l_{w}$	**CSAR**	${P/A}_{w}f_{c,0}^{\prime }$
		(m)	(mm)	(mm)	(%)		(%)
**Reference wall**	1	15.24	3048	304.8	15	10	0.1
**t**_**w**_	2	15.24	3048	203.2	15	15	0.1
	3	15.24	3048	406.4	15	7.5	0.1
$l_{\mathrm{be}}/l_{w}$	4	15.24	3048	304.8	10	10	0.1
	5	15.24	3.048	304.8	20	10	0.1
**CSAR**	6	15.24	1524	304.8	15	5	0.1
	7	15.24	4572	304.8	15	15	0.1
**ρ**_**be**_	8	15.24	3048	304.8	15	10	0.1
	9	15.24	3048	304.8	15	10	0.1
	10	15.24	3048	304.8	15	10	0.1
	11	15.24	3048	304.8	15	10	0.1
**ρ**_**web**_	12	15.24	3048	304.8	15	10	0.1
	13	15.24	3048	304.8	15	10	0.1
	14	15.24	3048	304.8	15	10	0.1
**s**	15	15.24	3048	304.8	15	10	0.1
	16	15.24	3048	304.8	15	10	0.1
${P/A}_{w}f_{c,0}^{\prime }$	17	15.24	3048	304.8	15	10	0.02
	18	15.24	3048	304.8	15	10	0.05
	19	15.24	3048	304.8	15	10	0.15
	20	15.24	3048	304.8	15	10	0.2
	21	15.24	3048	304.8	15	10	0.25

**Table 2:** Wall reinforcement detailing.

	***Wall ID***	**BE**^**1**^	**Web**^**2**^	**A**_**sb**_	**A**_**sw**_	**A**_**conf**_	**s**
				(mm^2^)	(mm^2^)	(mm^2^)	mm
**Reference wall**	1	1	1	284	129	129	102
**t**_**w**_	2	2	2	252	135	102	102
	3	3	3	302	135	135	102
$l_{\mathrm{be}}/l_{w}$	4	4	1	252	135	117	102
	5	5	1	252	135	121	102
**CSAR**	6	6	4	284	118	120	102
	7	7	1	283	133	132	102
**ρ**_**be**_	8	1	1	129	129	129	102
	9	1	1	200	129	129	102
	10	1	1	387	129	129	102
	11	1	1	509	129	129	102
**ρ**_**web**_	12	1	1	284	71	129	102
	13	1	1	284	200	129	102
	14	1	1	284	284	129	102
**s**	15	1	1	284	129	129	152
	16	1	1	284	129	129	203
${P/A}_{w}f_{c,0}^{\prime }$	17	1	1	284	129	129	102
	18	1	1	284	129	129	102
	19	1	1	284	129	129	102
	20	1	1	284	129	129	102
	21	1	1	284	129	129	102

Note: 1. See Fig. 1.

2. See Fig. 2.

**Fig. 3:**
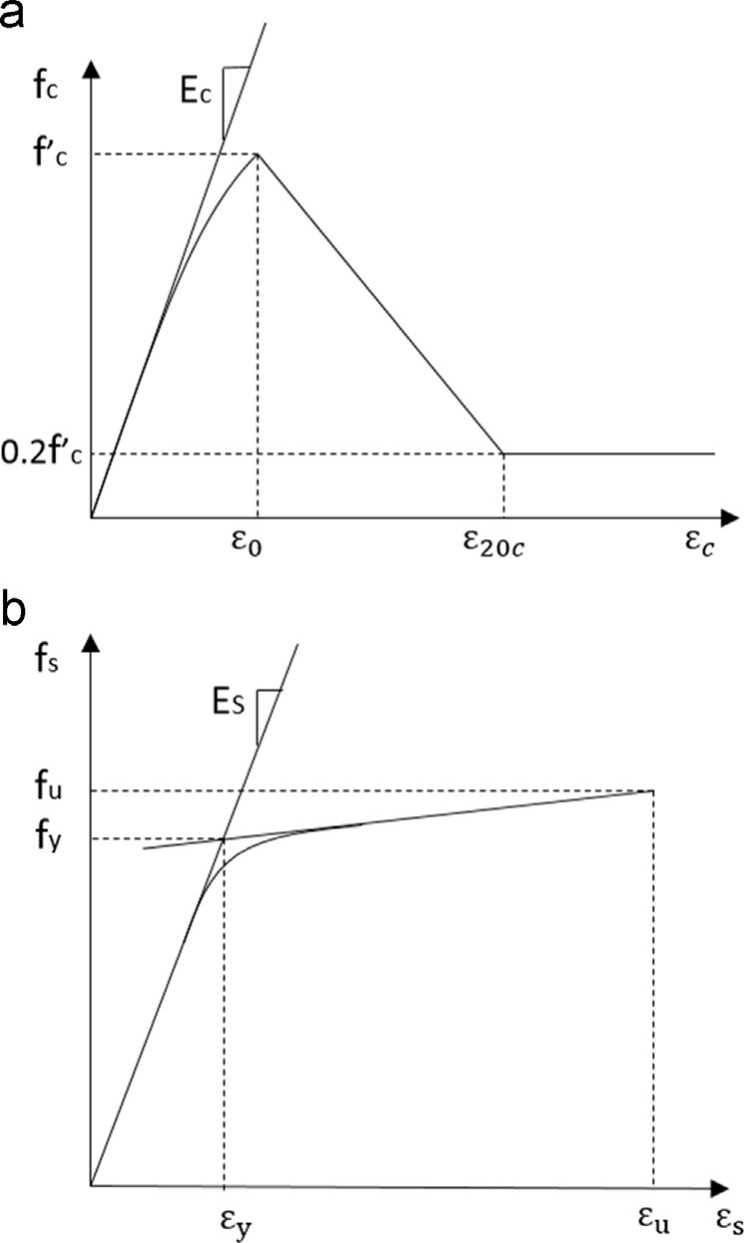
Material stress–strain curves for (a) concrete and (b) steel.

The same materials were used for all walls. Concrete compressive strength was 35 MPa (5000 psi), with siliceous aggregate and the stress–strain response shown in Fig. 3a. Confinement effects were modeled using the Chang and Mander confinement model [3]. Reinforcement had a yield strength of 414 MPa (60 ksi), with the stress–strain response shown in Fig. 3b and a fracture strain of 0.2.

**Fig. 4:**
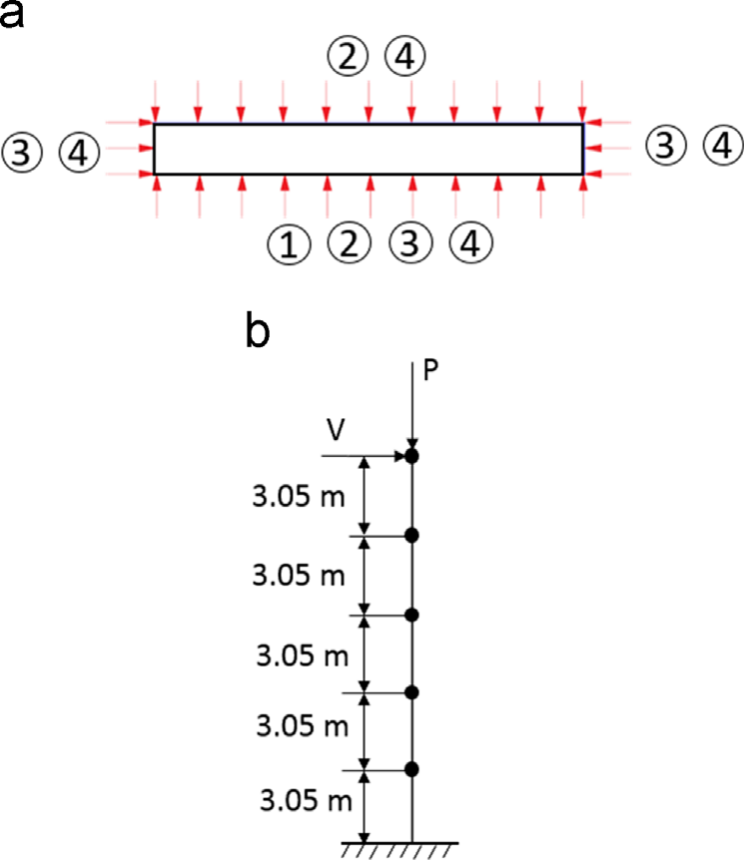
Boundary conditions for (a) thermal model (b) seismic model.

For each wall, four thermal boundary conditions, shown in Fig. 4a, were applied using SAFIR [4], each with a different number of sides of the first story exposed to fire. The ASTM E119 fire curve [5] was used, with durations of 0.25, 0.5, 1, 2, 3, and 4 h prior to a cooling phase of 5 °C/min. The fire-exposed sides of the wall were subject to radiation (emissivity coefficient *ε_r_*, 0.7) and convection (film coefficient h_c,_ 25 W/m^2^ °C) [6], [7]; the unexposed sides were subject to room temperature (film coefficient h_c_, 9 W/m^2^ °C) [7]. The thermal properties of the concrete and steel material models (conductivity and specific heat) were based on the material models in EC2-04 [6] built-in to SAFIR. Steel utilized the STEELEC2EN material model [4]. Concrete utilized the SILICON_ETC material model [8] for siliceous aggregate concrete, with concrete density of 2300 kg/m^3^ and moisture content of 46 kg/m^3^.

The seismic boundary conditions are shown in Fig. 4b. The walls were five stories with 3.05 m tall stories and a fixed base. Models were created in OpenSees [9], using force-based beam-column elements. Material regularization was used to eliminate mesh dependency [10]. Axial load was applied at the top of the wall. Lateral loads were applied at the top floor only. A reverse cyclic displacement history consisted of two cycles at each drift (0.01%, 0.02%, 0.05%, 0.1%, 0.2%, 0.3%, 0.4%, 0.5%, 0.75%, 1%, 1.5%, 2%, 2.5%, 3%, 3.5%, 4%, 4.5%, 5%).

## 3 Local data

Local data are the data for each material fiber in the lowest cross-section of the wall due to thermal and mechanical load. Five files are provided for no heat (Fiber_no_fire.xlsx), 1-sided fire (Fiber_1_side.xlsx), 2-sided fire (Fiber_2_side.xlsx), 3-sided fire (Fiber_3_side.xlsx), and 4-sided fire (Fiber_4_side.xlsx). Each tab provides data for one wall, identified by the Wall ID indicated in Table 1, Table 2.

**Fig. 5:**
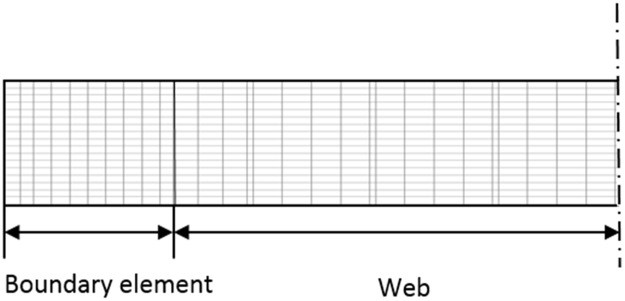
Mesh of concrete one-half of reference wall cross-section.

Each row provides data for a single fiber. The first five columns provide a fiber ID, the type of material (unconfined concrete, confined concrete and steel), fiber area, and coordinates of the fiber centroid. The mesh for one-half of the reference wall is shown in Fig. 5. Fifteen elements were used along the thickness of the wall and 63 elements were used along the length of the wall. Other walls were meshed to provide similar fiber sizes.

**Fig. 6:**
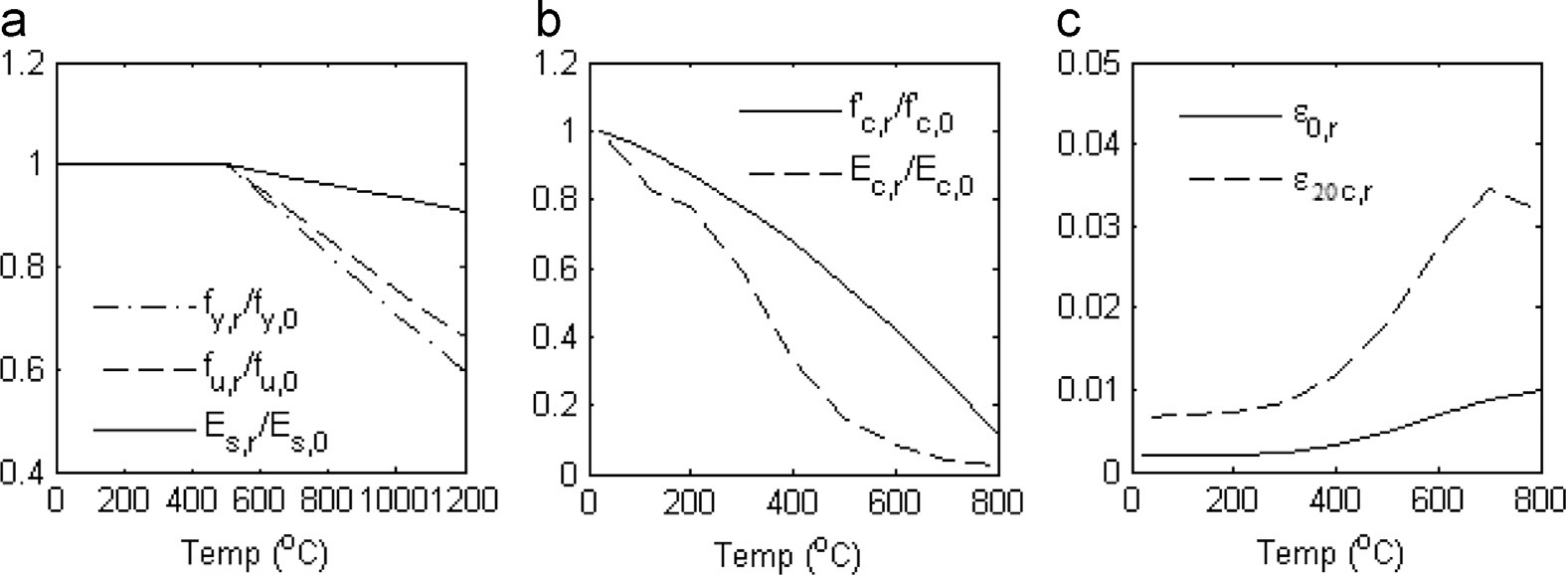
Variation of residual material properties with temperature: (a) Steel stiffness and strength [11]; (b) concrete stiffness and strength [12]; (c) concrete peak strain and crushing strain [12].

The subsequent columns in each local data file provide, for each fire duration 1) maximum historical temperature during the full heating-cooling cycle, 2) post-thermal residual material stiffness (*E*_*c*_ or *E*_*s*_) and material strength (*f’*_*c*_ or *f*_*y*_), and 3) damage ratio following seismic analysis. Post-thermal material properties were established from the factors shown in Fig. 6. Damage ratios were calculated as:

(1) $$\mathrm{Damage ratio}=\left(\varepsilon_{\max}-\varepsilon_{0}\right)/\left(\varepsilon_{20c}-\varepsilon_{0}\right)$$

where *ε*_*ma*x_ is the maximum measured compressive strain in the concrete fiber and all other parameters are shown in Fig. 3.

## 4 Global data

Five files (Envelope_no_fire.xlsx, Envelope_1_side.xlsx, Envelope_2_side.xlsx, Envelope_3_side.xlsx and Envelope_4_side.xlsx) provide data about load-deformation envelopes of walls. Each tab provides data for one wall, identified by the Wall ID in Tables 1 and 2.

Response_Quantities.xlsx provides data characterizing the backbone curves. Each tab provides data for one of the following response quantities: stiffness (*K*), maximum lateral load (*V_max_*), drift at failure (*Δ_fail_*), and the curvature at failure (*φ_fail_*). The stiffness is calculated at 75% of *V_max_*. The failure drift is the point when the load drops to 0.8*V_max_* or the point immediately before the lateral load decreases dramatically. The curvature at failure is taken at the same time as the failure drift, and is the curvature at the lowest integration point in the wall. Each row provides data for one wall, identified by the Wall ID indicated in Table 1, Table 2.
